# Inverse Relationship of Serum Hepcidin Levels with CD4 Cell Counts in HIV-Infected Patients Selected from an Indonesian Prospective Cohort Study

**DOI:** 10.1371/journal.pone.0079904

**Published:** 2013-11-11

**Authors:** Rudi Wisaksana, Quirijn de Mast, Bachti Alisjahbana, Hadi Jusuf, Primal Sudjana, Agnes R. Indrati, Rachmat Sumantri, Dorine Swinkels, Reinout van Crevel, Andre van der Ven

**Affiliations:** 1 Department of Internal Medicine Faculty of Medicine, Padjadjaran University/Hasan Sadikin Hospital, Bandung, Indonesia; 2 Health Research Unit, Faculty of Medicine, Padjadjaran University, Bandung, Indonesia; 3 Department of Internal Medicine, Radboud University Nijmegen Medical Centre, The Netherlands; 4 Clinical Pathology Faculty of Medicine, Padjadjaran University/Hasan Sadikin Hospital, Bandung, Indonesia; 5 Clinical Chemistry, Radboud University Nijmegen Medical Centre, The Netherlands; The University of Hong Kong, China

## Abstract

**Background:**

Distortion of iron homeostasis may contribute to the pathogenesis of human immunodeficiency virus (HIV) infection and tuberculosis (TB). We studied the association of the central iron-regulatory hormone hepcidin with the severity of HIV and the association between hepcidin and other markers of iron homeostasis with development of TB.

**Methods:**

Three groups of patients were selected from a prospective cohort of HIV-infected subjects in Bandung, Indonesia. The first group consisted of HIV-infected patients who started TB treatment more than 30 days after cohort enrollment (cases). The second group consisted of HIV-infected patients who were matched for age, gender and CD4 cell count to the cases group (matched controls). The third group consisted of HIV-infected patients with CD4 cell counts above 200 cells/mm^3^ (unmatched controls). Iron parameters including hepcidin were compared using samples collected at cohort enrollment, and compared with recently published reference values for serum hepcidin.

**Results:**

A total of 127 HIV-infected patients were included, 42 cases together with 42 matched controls and 43 unmatched controls. Patients with advanced HIV infection had elevated serum hepcidin and ferritin levels. Hepcidin levels correlated inversely with CD4 cells and hemoglobin. Cases had significantly higher hepcidin and ferritin concentrations at cohort enrollment compared to matched controls, but these differences were fully accounted for by the cases who started TB treatment between day 31 and 60 after enrollment. Hepcidin levels were not different in those with or without hepatitis C infection.

**Conclusion:**

Iron metabolism is distorted in advanced HIV infection with CD4 cell counts correlating inversely with serum hepcidin levels. High serum hepcidin levels and hyperferritinemia were found in patients starting TB treatment shortly after cohort enrollment, suggesting that these parameters have a predictive value for development of manifest TB in HIV-infected patients.

## Introduction

Alterations in iron distribution are common in infectious diseases and many of these alterations may be attributable to actions of the iron-regulatory hormone hepcidin [1]. Hepcidin degrades the sole cellular iron exporter ferroportin leading to reduced iron absorption in the intestine and iron retention in monocytes and macrophages and the spleen [2].

Changes in iron homeostasis have been described in HIV-infected patients. Epidemiological studies have found an association between elevated iron status, HIV progression and the risk for opportunistic infections [3], [4]. HIV replication involves several iron-dependent steps [5], [6], and as a central determinant of macrophage iron contents, hepcidin may play a distinct role in HIV pathogenesis. Indeed, hepcidin was recently shown to increase HIV-1 transcription in cultured monocytes and T-cells by degradation of ferroportin with a secondary increase in intracellular iron [7]. Hepcidin may also be involved in two important complications of human immunodeficiency virus infection/acquired immune deficiency syndrome (HIV/AIDS). First, elevated hepcidin levels limit iron supply to the bone marrow. This may contribute to HIV-associated anemia, which is a common complication of advanced HIV infection with negative impact on clinical outcome and quality of life [8]–[11]. Second, hepcidin-mediated iron accumulation in macrophages may increase the risk for outgrowth of intracellular pathogens like *Mycobacterium tuberculosis*. Worldwide, tuberculosis (TB) is the most important infectious disease complication among HIV-infected patients, and several lines of evidence suggest that macrophage iron content is related to the risk for developing active TB [12], [13]. Interestingly, hepcidin itself has antibacterial activity and hepcidin was shown to inhibit *M. tuberculosis* growth in vitro [14].

Data on hepcidin levels in HIV infected patients are rarely reported and were found to be related to ferroportin mutations (15). Apart from that, the pro-inflammatory cytokine interleukin (IL)-6 is a dominant regulator of hepatic hepcidin production in bacterial infections and other inflammatory conditions, but IL-6 concentrations are often only mildly elevated in viral infections. Indeed, recent studies have shown that hepcidin levels are reduced in hepatitis C virus (HCV) infection, which may contribute to pathological liver iron storage in patients with chronic HCV infection [16], [17].

The present study was performed in Indonesia, which has one of the fastest growing HIV epidemics in Asia with a high rate of TB and hepatitis C co-infection. Our primary aim was to study the effect HIV infection on serum hepcidin levels and other markers of iron homeostasis and to compare hepcidin levels with recently determined reference levels for hepcidin in healthy Dutch volunteers [18]. The secondary aim was to identify whether hepcidin and other markers of iron homeostasis were associated with development of TB more than 30 days after inclusion in the study. Finally, we studied whether iron parameters were influenced by factors such as gender, anemia, the use and kind of antiretroviral treatment (ART) and HCV co-infection.

## Methods

### Patients and setting

This study was designed as a nested case control study in a cohort of HIV-infected patients in Hasan Sadikin Hospital in Bandung, the referral hospital for HIV care in West-Java (11). Free anti-retroviral treatment (ART) is delivered since December 2004. Following the 2006 World Health Organization (WHO) guidelines [19], indications for start of ART during the study period were: a) HIV stage IV irrespective of CD4 cell count; b) HIV stage III with a CD4 cell count below 350 cells/mm^3^; and c) HIV stage I or II with a CD4 cell count less than 200 cells/mm^3^. First-line ART includes the nucleoside reverse transcriptase inhibitors (NRTI') zidovudine, stavudine and lamivudine, and the non-nucleoside reverse transcriptase inhibitors (NNRTI') efavirenz and nevirapine.

Starting in September 2007, all patients, aged 14 years and above are being enrolled in a prospective cohort study. Baseline enrollment consists of a structured interview, physical examination, chest X-ray and blood examination with storage of serum at −80°C. The latter is performed within one week from initial presentation and enrollment in the cohort. Injecting drug use is an important route of HIV transmission in West Java and HCV serostatus is determined in all patients. At the start of the cohort in 2007, some of the enrolled patients were already under treatment in Hasan Sadikin hospital or another hospital. Following their enrollment in the cohort, patients were scheduled for monthly follow up visits.

At time of enrollment in the cohort, all HIV-infected patients are screened for active TB by assessment of symptoms, chest X-ray, and sputum microscopy when indicated. The diagnosis of TB follows national and WHO guidelines, which are based on a combination of clinical features, imaging studies and sputum acid-fast bacilli (AFB) smear. For research purposes, *M. tuberculosis* genotyping has been done in this setting using spoligotyping as previously described, revealing nontuberculous mycobacteria in <1% of patients [20]. For this study, sputum smear-positive pulmonary TB was defined as at least one sputum smear positive for acid fast bacilli (AFB). Sputum smear-negative pulmonary TB as a chest X-ray finding consistent with tuberculosis together with a lack of response to a trial of broad-spectrum antibiotics and at two or more negative AFB sputum smears, including early morning samples. Extra pulmonary TB was defined as a positive AFB smear or culture from appropriate biopsy material, histopathological findings consistent with tuberculosis, or clinical signs and symptoms consistent with extra-pulmonary tuberculosis [21]. For this study we only included patients who either had bacteriologically proven TB, or a clear response to TB treatment.

From September 2007 until August 2010, 1323 HIV-infected patients aged 14 years old and above entered the cohort. Patients with signs or symptoms of an opportunistic infection or abnormal Chest X-ray at the time of cohort enrollment (n = 516) and patients who started on anti-TB treatment within one month after cohort enrollment (n = 9) were excluded from this study. From the remaining 798 patients, 45 developed TB more than 1 month after cohort enrollment and 42 of them were included as cases in this study. The remaining three patients were excluded because no serum samples were available from them. The one-month interval was deliberately chosen to avoid delayed diagnosis of co-existing subclinical TB that might already have been present at time of cohort enrollment. Two control groups of HIV-infected patients without a previous diagnosis of TB and without TB during follow were selected from the remaining 753 patients of our prospective HIV cohort: one group matched for age, gender and CD4 cell count (‘matched controls’; n = 42), and one control group consisting of only moderately immunocompromised HIV-patients with a CD4 cell count >200 cells/mm^3^ (‘unmatched controls’; n = 43). From all included patients, a total number of 38 out of 127 (30%) were already on ART with a median duration of 455 days (IQR 75-1008 days). All participants provided written informed consent to participate in this study. This study was approved by the Hasan Sadikin Hospital ethical committee.

### Laboratory methods

Serum hepcidin and iron parameters were measured in archived serum collected at time of cohort enrollment in the Department of Laboratory Medicine of the Radboud University Medical Centre using a combination of weak cation exchange chromatography and time-of-flight mass spectrometry (TOF MS). This technique allows discrimination between the three naturally occurring isoforms of hepcidin (hepcidin-20, -22, and -25), of which only the 25 amino acid form can block the iron transporter ferroportin and is referred to as “bioactive” hepcidin. An internal standard (synthetic hepcidin-24; Peptide International Inc.) was used for quantification [22], [23]. Peptide spectra were generated on a Microflex LT matrix-enhanced laser desorption/ionization TOF MS platform (Bruker Daltonics). Serum hepcidin-25 (further referred to as hepcidin) concentrations were expressed in nanomoles. The lower limit of detection of this method was 0.5 nM; average coefficients of variation were 2.7% (intra-run) and 6.5% (inter-run). The median level of serum hepcidin-25 concentrations in healthy Dutch male adults is 4.5 nM, while the reference range (P2.5–P97.5) is <0.5–14.7 nM; for premenopausal women these values are 2.0 nM (<0.5–12.3) [17], [24].

Serum concentrations of total iron, total iron binding capacity (TIBC), transferrin saturation (TS), ferritin, sTfR and C-reactive protein (CRP) were determined as described previously [25]. The following reference ranges were used: serum iron: 10–25 µmol/L; transferrin saturation (TS): 30–60%; total iron binding capacity (TIBC): 45–80 µmol/L: serum ferritin: 15–280 µg/L for males and 6–80 µg/L for premenopausal females; soluble transferrin receptor (sTfR): 0.76–1.76 mg/L. For statistical analyses, the lower limit of detection for CRP (5 mg/L) was used in samples with values below this detection limit. A full blood count was measured on a Cell Dyne 3000 (Abbot) hematology analyzer. Anemia was defined according to World Health Organization/AIDS Clinical Trial Group (WHO/ACTG) criteria as a hemoglobin level below 13.0 g/dL for men and below 12.0 g/dL for women. Anti-hepatitis C antibodies were tested by an electrochemiluminescence assay (ECLIA; Elecsys, Roche Diagnostics); CD4-cell counts were determined by flow cytometry (BD Biosciences, Jakarta, Indonesia). Due to limited volume of archived samples, serum levels of ferritin and sTfR were available in only 107 patients and serum iron, TIBC, TS and CRP in 38 patients.

### Data analysis and statistics

Serum concentrations of hepcidin and other markers of iron homeostasis were compared between cases and the two control groups. Subsequently, the effect of HIV infection on iron homeostasis was analyzed by comparing iron parameters in matched and unmatched controls with recently determined reference levels of serum hepcidin and by determination of the correlation of serum hepcidin levels with CD4 cell count. Finally, in the whole group of HIV-infected patients, associations were examined between iron parameters and gender, anemia, ART and the presence of HCV antibodies. Data are presented as medians with interquartile range (IQR) unless otherwise stated. Paired analysis for cases and the matched control group was performed using the Wilcoxon signed-rank test for continuous variables and the Mc Nemar test for categorical variables. Unpaired comparisons between cases and unmatched controls and between matched and unmatched controls were performed using the Mann-Whitney test and Chi-squared used. Comparison of median hepcidin levels in our study population with reference values was performed using the Wilcoxon signed-rank sum test. A two-sided P value of less than 0.05 was considered statistically significant. All statistical analyses were done by software SPSS version 17.0.

## Results

### Patient characteristics

A total number of 127 HIV-infected patients were included in our study: 42 cases who developed TB during follow-up and two control groups, consisting of 42 controls matched to cases for age, sex and CD4 cell count (‘matched controls’), and 43 unmatched controls with a CD4 cell count of more than 200 cells/mm3 (‘unmatched controls’). The majority of cases and matched controls had an advanced HIV infection with a median CD4 cell count of 30 cells/mm^3^ and 70 cells/mm^3^, respectively; the unmatched controls were moderately immunocompromised with a median CD4 cell count of 365 cells/mm^3^. Matched analysis did not found any difference between cases and matched control group for age, sex and CD4. Table 1 summarizes clinical and routine laboratory characteristics in study subjects at the time of cohort enrollment. In the cases, TB treatment was started at a median of 125 days (IQR 44–238 days) after cohort enrollment. Thirty-three patients (78%) of the cases had pulmonary TB; including 12 patients with AFB smear positive TB. Patients without bacteriological confirmation had a good response to TB treatment (by definition). Nine patients were diagnosed with extra-pulmonary TB of whom seven with lymphadenitis. The diagnosis of extra-pulmonary TB was made by histopathology examination in three cases and by a combination of clinical and radiological features, combined with a good response to anti-TB treatment in the remaining six. Ten of the cases had a history of TB-treatment, a median of 512 days (IQR 357–587 days) before enrollment which only one case did not finished 6 months TB treatment due to side effect. By definition, the patients in the control groups had neither a history of TB, nor any signs or symptoms suggesting development of active TB. After cohort enrollment, 27 out of 31 cases and 20 out of 31 matched controls initiated ART. CRP levels were only moderately elevated and mostly limited to the severely immunocompromised patients in the cases and matched controls group.

**Table 1:** Characteristics of HIV-infected patients who were diagnosed with tuberculosis after cohort enrollment and control groups.

	Cases^a^	Matched controls	*P* ^b^	Unmatched controls	*P* ^c^	*P* ^d^
	n = 42	n = 42		n = 43		
Age, years	29 (26–33)	30 (28–32)	0.51^e^	27 (25–29)	0.03	0.002
Male, %	90.5	90.5	1.00^f^	76.7	0.95	0.95
BMI, kg/m^2^	18.5 (16.9–21.1)^34^	18.1 (17.3–20.2)^30^	0.73	20.5 (18.8–21.8)^37^	0.016	0.005
Follow-up after cohort enrollment, days	836 (273–1110)	657 (68–1091)	0.257	539 (55–1095)	0.20	0.91
ART at baseline, %	28.6	26.2	0.81	34.9	0.53	0.38
History of TB treatment before initial visit, %	23.8	0	-	0	-	
CD4 cell count, cells/mm^3^	30 (14–138)	70 (27–137)	0.50^e^	365 (324–461)	<0.001	<0.001
CD4 cell count <200 cells/mm^3^	81.0	90.5	0.21	0	<0.001	<0.001
Hemoglobin, g/dL	12.0 (10.1–14.1)	13.7 (12.1–14.6)	0.02	14.5 (12.9–15.3)	<0.001	0.02
Anemia, %§	63.4	38.1	0.02	14.0	<0.001	0.01
C-reactive protein, mg/L	16.0 (5.0–36.0)^7^	5.0 (5.0–15.5)^16^	0.21	5.0 (5.0–5.0)^15^	0.02	0.20
C-reactive protein >10 mg/L, %	57.1	37.5	0.38	6.7	0.009	0.04
Alanine transaminase, IU/L	33.5 (20.0–54.5)	33.0 (20.8–66.0)	0.90	47.0 (21.0–69.0)	0.47	0.47
Random blood glucose, mg/dL	102.0 (87.3–116.5)^40^	92.5 (82.3–103.8)^40^	0.04	91.0 (86.8–98.0)^38^	0.03	0.76
Total cholesterol, mg/dL	158.5 (120.5–183.5)^26^	129.0 (111.0–167.0)^19^	0.30	158.0 (120.5–178.5)^21^	0.89	0.33
LDL, mg/dL	84.5 (63.5–114.0)^26^	70.0 (49.0–100.0)^19^	0.24	78.0 (54.5–103.5)^21^	0.76	0.52
Triglyceride, mg/dL	152.5 (103.0–196.0)^26^	145.0 (114.0–172.0)^19^	0.77	113.0 (96.5–154.0)^21^	0.13	0.11
Hepatitis C antibodies present, %†	72.5^40^	73.7^38^	0.91	66.7^36^	0.58	0.51

Data are presented as medians and interquartile range (IQR) unless stated otherwise. ART, antiretroviral therapy; BMI, body mass index; LDL, low-density lipoprotein.

The number of samples available in each group is shown in superscript after the IQR or percentage if less than total number.

a Cases group: HIV-infected patients diagnosed with tuberculosis (TB) more than 1 month after cohort enrollment. Matched controls: HIV-infected patients without diagnosis of TB before or after cohort enrollment, matched for age, gender and CD4 cell count to cases group. Unmatched controls: HIV-infected patients without diagnosis of TB before or after cohort enrollment with CD4 cells >200 cells/mm^3^. *P* values were determined for ^b^ difference between matched controls and cases, ^c^ between unmatched controls and cases and. ^d^ between matched and unmatched controls.

e Wilcoxon signed rank test.

f Mc Nemar test.

§ Anemia defined as hemoglobin level <13 g/dL for men or <12 g/dL for women.

† Defined as positive anti-HCV antibodies.

### Iron parameters in relation to HIV status and CD4 cell count

**Figure 1:**
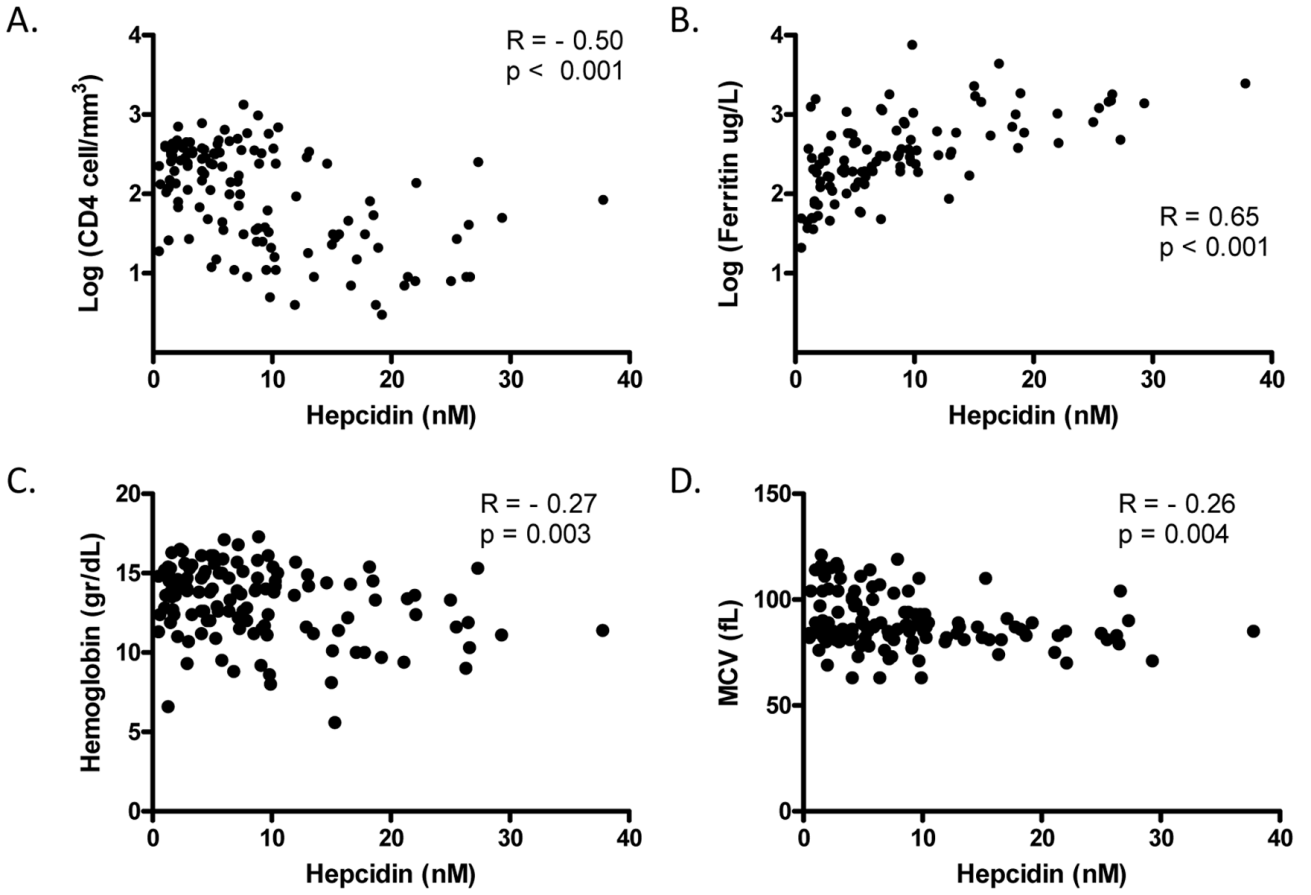
Correlation of serum hepcidin levels with hematology and iron parameters. Spearman correlation coefficient of serum hepcidin levels with (A) CD4 cell count, (B) serum ferritin levels, (C) hemoglobin levels and (D) mean corpuscular volume (MCV).

Median serum hepcidin levels in the whole group of HIV-infected patients were above reference values for healthy Dutch adult males (7.3 vs. 4.5 nM; p<0.001) and premenopausal females (3.1 vs. 2.0 nM; p = 0.028) [16], [22]. However, these higher hepcidin were largely restricted to cases and matched controls, i.e. those with advanced HIV infection (table 2). The unmatched male controls had a similar median hepcidin level value as healthy Dutch adults (4.4 [2.1–8.1] nM), but female unmatched controls had a higher median hepcidin level (4.4 [1.5–8.2] nM). The higher hepcidin levels in cases compared to unmatched controls were associated with higher serum levels of the iron storage protein ferritin and lower serum iron, TIBC and mean corpuscular volume (MCV) values (table 2). Serum hepcidin levels also correlated inversely with CD4 cell counts, hemoglobin levels and MCV, and positively with serum ferritin levels (Fig. 1A–D).

**Table 2:** Hepcidin and iron parameters at cohort enrollment in HIV-infected patients with (cases) or without (controls) incident TB.

	Cases group^a^	Matched controls	*P* ^b^	Unmatched controls	*P* ^c^	*P* ^d^
	n = 42	n = 42		n = 43		
Hepcidin, nM	9.9 (5.0–18.2)	7.2 (2.9–10.2)	0.035	4.4 (2.0–7.9)	<0.001	0.06
Ferritin, µg/l	680.5 (245.0–1439.0)^34^	297.0 (175.0–462.0)^37^	0.006	213.5 (74.3–358.0)^36^	<0.001	0.09
sTfR, mg/l	1.4 (1.1–2.2)^34^	1.4 (1.2–1.9)^37^	0.61	1.3 (1.1–1.6)^36^	0.13	0.28
Serum iron, µmol/l	7.0 (5.0–11.0)^7^	10.5 (6.3–17.5)^16^	0.27	16.0 (10.0–27.0)^15^	0.03	0.13
TIBC, µmol/l	39.0 (38.0–54.0)^7^	51.0 (43.3–59.8)^16^	0.26	57.0 (49.0–66.0)^15^	0.03	0.08
Transferrin saturation, %	16.0 (12.0–27.0)^7^	24.0 (14.5–31.3)^16^	0.26	26.0 (16.0–47.0)^15^	0.15	0.51
MCV, fl	83.5 (80.3–92.5)^39^	85.5 (82.0–93.8)	0.53	88.0 (84.0–104.0)	0.049	0.11
MCH, pg	29.0 (26.3–32.0)^39^	29.0 (28.0–32.0)	0.98	30.0 (28.0–35.0)	0.14	0.14

Data depicted are median with interquartile range (IQR). TB, tuberculosis; TIBC, total iron binding capacity; sTfR, soluble transferrin receptor; MCV, mean cell volume; MCH, mean cell hemoglobin.

Numbers of samples available in each group is shown in superscript after the IQR if less than total number.

a Cases group: HIV-infected patients diagnosed with tuberculosis (TB) more than 1 month after initial visit. Matched controls: HIV-infected patients without diagnosis of TB before or after initial visit, matched for age, gender and CD4 cell count to cases group. Unmatched controls: HIV-infected patients without diagnosis of TB before or after initial visit not matched for age, gender and CD4 cell count to cases group. *P* values were determined for ^b^ difference between matched controls and cases by using the Wilcoxon signed-rank test and ^c^ between unmatched controls and cases and ^d^ matched and unmatched controls by using the Mann-Whitney test.

### Iron parameters and development of TB

Compared to matched controls, cases starting TB treatment (‘cases’) more than 30 days after enrollment had significantly higher serum hepcidin (p = 0.035) and ferritin levels (p = 0.006)(Table 2). However, this difference was fully accounted for by the cases starting TB treatment between day 31 and 60. Compared to those starting TB treatment more than 60 days after cohort enrollment, the cases had significantly higher serum hepcidin and ferritin levels at cohort enrollment with median (IQR) serum levels for hepcidin of 16.1 nM (8.4–20.6 nM) vs. 5.8 nM (4.1–14.1 nM; p = 0.01) and for ferritin of 1206 µg/l (591–1799 µg/l) vs. 310 µg/l (190–887 µg/l; p = 0.004), despite the fact that clinical and radiological examination showed no signs for TB at cohort enrollment. There were no significant differences in either hepcidin or other iron indicators between cases starting TB treatment more than 60 days after cohort enrollment and their matched controls. Combining all cases of pulmonary TB, smear positive pulmonary TB was associated with a significantly higher serum hepcidin level at cohort enrollment than smear negative TB (15.9 nM [13.1–21.9 nM] vs. 5.3 nM [4.1–5.5 nM] nM; p = 0.003). In addition, cases developing extrapulmonary TB had significantly higher ferritin levels at cohort enrollment than cases with pulmonary TB (1351 µg/L [725–1800 µg/L] vs. 491 µg/L [193–1250 µg/L]; p = 0.03), but similar hepcidin levels (9.4 nM [6.0–11.8 nM] vs. 11.5 nM [4.9–17.0 nM]; p = 0.47).

### Other factors affecting iron parameters

Apart from HIV and/or TB infection, iron parameters may be influenced by other factors such as gender, anemia, the use and kind of ART and HCV co-infection. As shown in Table 3, women had significantly lower serum hepcidin and ferritin levels than men (p<0.01) owing to their lower total iron stores. Second, anemic patients had higher ferritin and hepcidin levels (p<0.001 and p = 0.05, respectively) together with a lower value of TIBC (p<0.001), which is consistent with anemia of chronic disease. Third, patients taking stavudine had somewhat higher hepcidin concentrations than those taking zidovudine, although this difference was not statistically significant (p = 0.24). Finally, HCV-infected patients had slightly higher serum hepcidin, ferritin and transferrin saturation levels, although these differences were not statistically significant.

**Table 3:** Hepcidin, iron and inflammatory parameters in HIV-infected patients at cohort enrollment.

Characteristics	Hepcidin	Serum iron	TIBC	Transferrin	Ferritin	sTfR	CRP
	(nM)	(µmol/l)	(µmol/l)	saturation (%)	(µg/l)	(mg/l)	(mg/L)
Gender							
Male	7.3 (4.0–13.1)^109^	11.5 (6.3–20.0)^28^	53.5 (39.0–62.8)^28^	27.5 (14.5–36.5)^28^	344 (194–795)^92^	1.4 (1.2–2.0)^92^	5.0 (5.0–16.0)^28^
Female	3.1 (1.5–6.9)^18^ a	9.5 (8.5–12.5)^10^	54.0 (48.5–66.8)^10^	17.0 (12.8–25.3)^10^	60 (46–132)^15^ a	1.5 (1.1–1.9)^15^	5.0 (5.0–7.3)^10^
Anemia							
Yes	9.2 (4.6–16.4)^49^	9.0 (4.5–13.0)^17^	42.0 (38.5–49.0)^17^	21.0 (11.0–33.0)^17^	698 (257–1460)^43^	1.4 (1.0–2.2)^43^	14.0 (5.0–41.5)^17^
No	5.3 (2.5–9.6)^78^ b	16.0 (9.0–20.0)^21^	61.0 (55.0–66.0)^21^ b	26.0 (16.0–31.0)^21^	217 (112–351)^64^ b	1.4 (1.2–1.8)^64^	5.0 (5.0–5.0)^21^ b
Receive ART							
Yes	5.6 (2.6–9.8)^38^	20.5 (9.8–36.8)^9^	45.5 (39.0–53.0)^9^	42.5 (19.0–87.0)^9^	290 (161–1081)^32^	1.7 (1.3–2.2)^32^	7.0 (5.0–26.5)^9^
ZDV containing	5.2 (2.2–9.6)^26^	27.0 (4.0–40.0)^7^	49.0 (39.0–57.0)^7^	47.0 (10.0–93.0)^7^	252 (130–741)^22^	1.7 (1.2–2.1)^22^	7.0 (5.0–14.0)^7^
d4T containing	7.4 (3.2–16.5)^12^	13.0 (12.0–14.0)^2^	40.5 (31.0–50.0)^2^	33.0 (28.0–38.0)^2^	431 (205–1227)^10^	1.8 (1.3–2.7)^10^	22.0 (5.0–39.0)^2^
No	7.2 (3.3–12.5)^89^	10.5 (6.8–18.0)^29^	55.5 (47.8–65.3)^29^	19.0 (13.0–28.3)^29^ c	301 (166–608)^75^	1.3 (1.1–1.7)^75^ b	5.0 (5.0–12.5)^29^
HCV							
Yes	6.8 (3.0–11.6)^80^	11.0 (6.0–11.0)^19^	55.0 (39.0–64.0)^19^	27.0 (16.0–32.0)^19^	298 (191–596)^66^	1.3 (1.1–1.9)^66^	5.0 (5.0–16.0)^19^
No	5.8 (2.5–13.1)^33^	9.0 (7.0–12.0)^11^	50.0 (44.0–56.0)^11^	16.0 (13.0–25.0)^11^	257 (84–698)^29^	1.5 (1.2–2.2)^29^	5.0 (5.0–14.0)^11^

Data are based on 127 HIV-infected patients belonging to case group and the matched and unmatched control groups. Data depicted as median with interquartile range. TIBC, total iron binding capacity; sTfR, soluble transferrin receptor; CRP, C-reactive protein; ART, antiretroviral therapy; HCV, hepatitis C; ZDV, zidovudine; d4T, stavudine.

Hemoglobin and serum hepcidin levels were available for 127 patients; ferritin and sTfR for 107 patients and serum iron, TIBC, transferrin saturation and CRP in 38 patients, Anti HCV antibodies were available in 113 patients. Numbers of samples per groups are shown in superscript after the IQR.

a p<0.01 between male and female group;

b p<0.01 between anemia and non-anemia group;

c p<0.05 between group with and without ART.

## Discussion

Different studies have shown that disturbances in iron homeostasis and anemia are associated with advanced HIV infection and an adverse outcome [3], [4], [9], [26]. To our knowledge, only one study has so far reported data on hepcidin in HIV-infected patients. Masaisa et al. found a mutation (Q248H) in the iron exporter ferroportin to be related to lower serum hepcidin levels, higher ferritin levels and a higher risk for pulmonary TB [15]. Our data show that serum hepcidin levels were increased in Indonesian patients with advanced HIV infection. In contrast, HIV-infected patients without TB and with CD4 cell counts above 200 cells/mm^3^ (unmatched controls) had similar serum hepcidin levels as healthy Dutch adults. Inflammatory cytokines, especially IL-6, are important inducers of hepatic hepcidin expression. Our observation of an inverse relation of CD4 cell counts with serum hepcidin levels and the fact that elevated CRP levels were more common in those with advanced HIV-infection support the notion that elevated hepcidin levels in advanced HIV are predominantly caused by HIV-associated inflammation. Moreover, only few of the unmatched controls with CD4 cell counts above 200 cells/mm^3^ had an elevated CRP level (6.7%), and this may explain why this group had similar hepcidin levels as healthy Dutch individuals. Other HIV-specific mechanisms, apart from inflammation, may also contribute to higher hepcidin levels. HIV can directly infect bone marrow progenitor cells leading to bone marrow suppression, which is associated with upregulated hepcidin expression [27], [28]. Zidovudine is also known for its suppressive effect on the bone marrow, but use of zidovudine was not associated with higher hepcidin levels in our study.

Our finding of increased hepcidin expression with secondary iron dysregulation in advanced HIV infection may have several clinical consequences. First, increased hepcidin expression leads to macrophage iron loading and this may be associated with a higher risk for intracellular infections. *M. tuberculosis* intracellular growing bacterium that needs iron for its multiplication, and macrophage iron loading might thus stimulate development of TB. In our present study, we indeed found higher serum hepcidin and ferritin levels at cohort enrollment in those who subsequently started TB treatment, but these higher levels were restricted to those starting treatment from day 31 to 60 after enrollment. Recent studies have shown that *M. tuberculosis* and mycobacterial components may also induce hepcidin and we speculate that the presence of subclinical TB at cohort enrollment accounted for these higher hepcidin and ferritin levels [14], [29]. Alternatively, higher hepcidin and ferritin levels may put HIV-infected patients at an increased risk for TB reactivation of TB immune reconstitution inflammatory syndrome (IRIS). The latter seems less likely, because hepcidin not only functions as an iron regulator, but also possesses antibacterial effects. Indeed, other authors have suggested increased hepcidin production in response to *M. tuberculosis* infection to represent a host defense mechanism [14], [29]. A second possible clinical consequence of the high hepcidin levels is its effect on HIV progression. Several lines of evidence suggest that iron accumulation in macrophages promotes HIV progression (reviewed in Drakesmith et al. [5] and currently by Nekhai et al. [6]). Hepcidin regulates macrophage iron content by binding to and degrading the sole iron exporter ferroportin [2], and a recent *ex vivo* study showed that hepcidin increases HIV transcription [7]. Chronic inflammation with prolonged hepcidin over-expression may thus explain earlier findings of a positive correlation between the degree of iron loading in macrophages and mortality [3]. Third, hepcidin has turned out to be a central mediator of the anemia of inflammation by reducing iron absorption from the intestine and iron recycling by macrophages of the spleen and the reticulo-endothelial system. Over-expression of hepcidin in transgenic mice indeed led to typical features of anemia of inflammation [30]. Hepcidin can also directly inhibit red blood cell production during inflammation [31]. Anemic patients in our study had significantly higher hepcidin levels and there was a negative correlation of hepcidin levels with hemoglobin levels. Thus, prolonged HIV-associated changes in absorption and distribution of iron through the actions of hepcidin are likely to contribute to the still poorly understood anemia of advanced HIV infection. For clinical management of anemic HIV-infected patients, our data suggest that iron supplementation will be of limited benefit as the elevated hepcidin levels compromise iron absorption. Serum hepcidin may prove a useful parameter to select those patients with absolute iron deficiency who may benefit from iron supplementation.

Serum ferritin levels were strongly elevated in patients who developed tuberculosis 31–60 days after enrollment. Several mechanisms may explain the high ferritin level in these patients. First of all, ferritin is an acute phase protein and inflammation can independently increase ferritin expression. Unfortunately, sufficient data to analyze this further were not available. Other factors that may contribute to high ferritin levels include HIV [32], chronic liver disease [33], metabolic syndrome or iron loading anemia. Some of these factors do not seem relevant: increased ferritin levels were neither noticed in control patients with an untreated HIV infection nor in those with HCV infection. Alcoholic liver diseases is also very rare in our Muslim population while none of the patients was known with an iron loading anemia or a history of recent blood transfusion. Although obesity and hypertension are rare in our population, a trend for higher triglyceride and random blood glucose levels was noticed in the cases, suggesting some metabolic disturbance. A fatty liver with hyperferritinemia can also be seen in other conditions such as rapid weight loss, starvation, and use of illicit drugs [34]. Interestingly, a large autopsy study from India revealed that tuberculosis was the second commonest cause of steatohepatitis [35] and severe hyperferritinemia in *Mycobacterium tuberculosis* infection has recently been reported, especially among HIV-infected patients [36]. Our results were however not obtained from patients with active TB but an average of 125 days earlier when no signs of an active (opportunistic) infection were present. Possibly, serum ferritin may thus be a useful and low-cost marker to predict the change for development of TB in near future. Hepcidin seems less useful for this purpose because of its high cost and limited availability of assays.

As injecting drug use is the main route of HIV transmission in the setting of this study, a significant proportion of patients were also infected with HCV. Chronic HCV infection is associated with disturbances in iron homeostasis, most prominently iron accumulation in hepatocytes [37]. Two recent studies found relatively low hepcidin levels in patients with HCV, suggesting that HCV impairs hepcidin production, which may in turn contribute to high iron contents of the liver [15], [16]. These findings could not be confirmed in our study, which is the first study to report data in HCV/HIV co-infected patients.

Our study has several limitations. First, in more than 50% in all cases there was no culture confirmation of TB. Unfortunately, it is well known that sputum microscopy and culture both suffer from decreased sensitivity in HIV-infected individuals [38], [39]. We like to point out that we only included patients with bacteriological proof or clinical and radiological signs of tuberculosis, plus a positive treatment response. Second, as also mentioned above, we cannot exclude with certainty that TB was already present in some of the cases during cohort enrollment, even though clinical and radiological examination failed to show any signs of active TB. The fact that as much as 85% of cases and matched controls had a CD4 count less than 200 cells/mm^3^ at cohort enrollment increases the risk for subclinical TB. Third, development of an IRIS against TB following the start of ART is common in this setting [40], and some of the patients starting TB treatment may in fact have suffered from TB IRIS. Fourth, data on serum iron, transferrin saturation and CRP were only available in a subset of patients due to limited volume of some of the archived samples, although no differences in clinical characteristics were found between patients with and without available samples (data not shown). Finally, we acknowledge that the number of patients developing TB was rather small despite the fact that 798 patients were prospectively followed for a median follow-up time of 836 days (table 1). The small sample size was also the reason for not performing a multivariate analysis. However, we feel that none of these issues can account for the significant associations we found between hepcidin and CD4 cell count and development of TB.

In conclusion, our study shows that advanced HIV infection is associated with increased hepcidin expression and characteristic features of iron maldistribution. This may, among others, stimulate HIV progression and contribute to HIV-associated anemia. Patients starting TB treatment shortly after cohort enrollment had significantly higher serum hepcidin and ferritin level at cohort enrollment, but this effect was not present in those starting TB treatment after at least 2 months.

## References

[1] Ganz T (2011) Hepcidin and iron regulation, 10 years later. Blood 117: 4425–4433. 21346250 10.1182/blood-2011-01-258467 PMC3099567

[2] Nemeth E, Tuttle MS, Powelson J, Vaughn MB, Donovan A, et al (2004) Hepcidin regulates cellular iron efflux by binding to ferroportin and inducing its internalization. Science 306: 2090–2093. 15514116 10.1126/science.1104742

[3] de Monye C, Karcher DS, Boelaert JR, Gordeuk VR (1999) Bone marrow macrophage iron grade and survival of HIV-seropositive patients. AIDS 13: 375–380. 10199228 10.1097/00002030-199902250-00010

[4] McDermid JM, Jaye A, Schim van der Loeff MF, Todd J, Bates C, et al (2007) Elevated iron status strongly predicts mortality in West African adults with HIV infection. J Acquir Immune Defic Syndr 46: 498–507. 18077841 10.1097/qai.0b013e31815b2d4b

[5] Drakesmith H, Prentice A (2008) Viral infection and iron metabolism. Nat Rev Microbiol 6: 541–552. 18552864 10.1038/nrmicro1930

[6] Nekhai S, Kumari N, Dhawan S (2013) Role of cellular iron and oxygen in the regulation of HIV-1 infection. Future Virol 8: 301–311. 23678366 10.2217/fvl.13.6 PMC3652425

[7] Xu M, Kashanchi F, Foster A, Rotimi J, Turner W, et al (2010) Hepcidin induces HIV-1 transcription inhibited by ferroportin. Retrovirology 7: 104. 21126372 10.1186/1742-4690-7-104 PMC3022686

[8] Belperio PS, Rhew DC (2004) Prevalence and outcomes of anemia in individuals with human immunodeficiency virus: a systematic review of the literature. Am J Med 116 Suppl 7A27S–43S. 15050884 10.1016/j.amjmed.2003.12.010

[9] Lundgren JD, Mocroft A (2003) Anemia and survival in human immunodeficiency virus. Clin Infect Dis 37 Suppl 4S297–S303. 14581998 10.1086/376909

[10] Volberding PA, Levine AM, Dieterich D, Mildvan D, Mitsuyasu R, et al (2004) Anemia in HIV infection: clinical impact and evidence-based management strategies. Clin Infect Dis 38: 1454–1463. 15156485 10.1086/383031

[11] Wisaksana R, Sumantri R, Indrati AR, Zwitser A, Jusuf H, et al (2011) Anemia and iron homeostasis in a cohort of HIV-infected patients in Indonesia. BMC Infect Dis 11: 213. 21827653 10.1186/1471-2334-11-213 PMC3199780

[12] Boelaert JR, Vandecasteele SJ, Appelberg R, Gordeuk VR (2007) The effect of the host' iron status on tuberculosis. J Infect Dis 195: 1745–1753. 17492589 10.1086/518040

[13] Baker MA, Wilson D, Wallengren K, Sandgren A, Iartchouk O, et al (2012) Polymorphisms in the Gene that encodes the iron transport protein ferroportin 1 influence susceptibility to tuberculosis. J Infect Dis 205: 1043–1047. 22357659 10.1093/infdis/jis026

[14] Sow FB, Florence WC, Satoskar AR, Schlesinger LS, Zwilling BS, et al (2007) Expression and localization of hepcidin in macrophages: a role in host defense against tuberculosis. J Leukoc Biol 82: 934–945. 17609338 10.1189/jlb.0407216

[15] Masaisa F, Breman C, Gahutu JB, Mukiibi J, Delanghe J, et al (2012) Ferroportin (SLC40AI) Q248H mutation is associated with lower circulating serum hepcidin levels in Rwandese HIV-positive women. Ann Hematol 91: 911–916. 22249207 10.1007/s00277-011-1400-3

[16] Fujita N, Sugimoto R, Takeo M, Urawa N, Mifuji R, et al (2007) Hepcidin expression in the liver: relatively low level in patients with chronic hepatitis C. Mol Med 13: 97–104. 17515961 10.2119/2006-00057.Fujita PMC1869620

[17] Girelli D, Pasino M, Goodnough JB, Nemeth E, Guido M, et al (2009) Reduced serum hepcidin levels in patients with chronic hepatitis C. J Hepatol 51: 845–852. 19729219 10.1016/j.jhep.2009.06.027 PMC2761995

[18] Galesloot TE, Vermeulen SH, Geurts-Moespot AJ, Klaver SM, Kroot JJ, et al (2011) Serum hepcidin: reference ranges and biochemical correlates in the general population. Blood 117: e218–e225. 21527524 10.1182/blood-2011-02-337907

[19] Gilks CF, Crowley S, Ekpini R, Gove S, Perriens J, et al (2006) The WHO public-health approach to antiretroviral treatment against HIV in resource-limited settings. Lancet 368: 505–510. 16890837 10.1016/S0140-6736(06)69158-7

[20] Parwati I, van Crevel R, van Soolingen D, van der Zanden A (2003) Application of spoligotyping to noncultured Mycobacterium tuberculosis bacteria requires an optimized approach. J Clin Microbiol 41: 5350–5351. 14605204 10.1128/JCM.41.11.5350-5351.2003 PMC262530

[21] Stop TB Department of HIV/AIDS WHO (2006) Improving the diagnosis and treatment of smear-negative pulmonary and extrapulmonary tuberculosis among adults and adolescents. Recommendations for HIV-prevalent and resource-constrained settings. Geneva: World Health Organization.

[22] Kroot JJ, Laarakkers CM, Geurts-Moespot AJ, Grebenchtchikov N, Pickkers P, et al (2010) Immunochemical and mass-spectrometry-based serum hepcidin assays for iron metabolism disorders. Clin Chem 56: 1570–1579. 20739637 10.1373/clinchem.2010.149187

[23] Swinkels DW, Girelli D, Laarakkers C, Kroot J, Campostrini N, et al (2008) Advances in quantitative hepcidin measurements by time-of-flight mass spectrometry. PLoS One 3: e2706. 18628991 10.1371/journal.pone.0002706 PMC2442656

[24] www.hepcidinanalysis.com, accessed June 2012.

[25] de Mast Q, Nadjm B, Reyburn H, Kemna EH, Amos B, et al (2009) Assessment of urinary concentrations of hepcidin provides novel insight into disturbances in iron homeostasis during malarial infection. J Infect Dis 199: 253–262. 19032104 10.1086/595790

[26] O'rien ME, Kupka R, Msamanga GI, Saathoff E, Hunter DJ, et al (2005) Anemia is an independent predictor of mortality and immunologic progression of disease among women with HIV in Tanzania. J Acquir Immune Defic Syndr 40: 219–225. 16186741 10.1097/01.qai.0000166374.16222.a2

[27] Kanda J, Mizumoto C, Kawabata H, Tsuchida H, Tomosugi N, et al (2008) Serum hepcidin level and erythropoietic activity after hematopoietic stem cell transplantation. Haematologica 93: 1550–1554. 18641032 10.3324/haematol.12399

[28] Pak M, Lopez MA, Gabayan V, Ganz T, Rivera S (2006) Suppression of hepcidin during anemia requires erythropoietic activity. Blood 108: 3730–3735. 16882706 10.1182/blood-2006-06-028787 PMC1895477

[29] Sow FB, Nandakumar S, Velu V, Kellar KL, Schlesinger LS, et al (2011) Mycobacterium tuberculosis components stimulate production of the antimicrobial peptide hepcidin. Tuberculosis (Edinb) 91: 314–321. 21482189 10.1016/j.tube.2011.03.003

[30] Roy CN, Mak HH, Akpan I, Losyev G, Zurakowski D, et al (2007) Hepcidin antimicrobial peptide transgenic mice exhibit features of the anemia of inflammation. Blood 109: 4038–4044. 17218383 10.1182/blood-2006-10-051755 PMC1874566

[31] Dallalio G, Law E, Means RT Jr (2006) Hepcidin inhibits in vitro erythroid colony formation at reduced erythropoietin concentrations. Blood 107: 2702–2704. 16332970 10.1182/blood-2005-07-2854 PMC1895381

[32] Drakesmith H, Chen N, Ledermann H, Screaton G, Townsend A, et al (2005) HIV-1 Nef down-regulates the hemochromatosis protein HFE, manipulating cellular iron homeostasis. Proc Natl Acad Sci U S A 102: 11017–11022. 16043695 10.1073/pnas.0504823102 PMC1180511

[33] Camaschella C, Poggiali E (2009) Towards explaining “nexplained hyperferritinemia”. Haematologica 94: 307–309. 19252171 10.3324/haematol.2008.005405 PMC2649347

[34] Hearnshaw S, Thompson NP, McGill A (2006) The epidemiology of hyperferritinaemia. World J Gastroenterol 12: 5866–5869. 17007054 10.3748/wjg.v12.i36.5866 PMC4100669

[35] Tripathi PB, Amarapurkar AD (2009) Morphological spectrum of gastrointestinal tuberculosis. Trop Gastroenterol 30: 35–39. 19624086

[36] Visser A, van de Vyver (2011) Severe hyperferritinemia in Mycobacteria tuberculosis infection. Clin Infect Dis 52: 273–274. 21288855 10.1093/cid/ciq126

[37] Silva IS, Perez RM, Oliveira PV, Cantagalo MI, Dantas E, et al (2005) Iron overload in patients with chronic hepatitis C virus infection: clinical and histological study. J Gastroenterol Hepatol 20: 243–248. 15683427 10.1111/j.1440-1746.2004.03549.x

[38] Chartier L, Leng C, Sire JM, Le Minor O, Saman M, et al (2011) Factors associated with negative direct sputum examination in Asian and African HIV-infected patients with tuberculosis (ANRS 1260). PLoS One 6: e21212. 21731675 10.1371/journal.pone.0021212 PMC3121737

[39] Lawn SD, Wood R (2011) Tuberculosis in antiretroviral treatment services in resource-limited settings: addressing the challenges of screening and diagnosis. J Infect Dis 204 Suppl 4S1159–S1167. 21996698 10.1093/infdis/jir411 PMC3192543

[40] Cohen K, Meintjes G (2010) Management of individuals requiring antiretroviral therapy and TB treatment. Curr Opin HIV AIDS 5: 61–9. 20046149 10.1097/COH.0b013e3283339309 PMC2936963

